# *B. adolescentis* ameliorates chronic colitis by regulating Treg/Th2 response and gut microbiota remodeling

**DOI:** 10.1080/19490976.2020.1826746

**Published:** 2021-02-09

**Authors:** Lina Fan, Yadong Qi, Siwen Qu, Xueqin Chen, Aiqing Li, Maher Hendi, Chaochao Xu, Lan Wang, Tongyao Hou, Jianmin Si, Shujie Chen

**Affiliations:** a Department of Gastroenterology, Sir Run Run Shaw Hospital, School of Medicine, Zhejiang University, Zhejiang, China; b Institute of Gastroenterology, Zhejiang University, Zhejiang, China; c Department of General Surgery, Sir Run Run Shaw Hospital, School of Medicine, Zhejiang University, Zhejiang, China

**Keywords:** Chronic colitis, *B. adolescentis*, Gut barrier, immunomodulation, microbiota

## Abstract

Inflammatory bowel disease (IBD) is defined as an immune dysregulation disease with poor prognosis. Various therapies based on gut microbe modulation have been proposed. In this study, we aim to explore the therapeutic effect of *B. adolescentis* on IBD, as well as the immune and microecology mechanism of *B. adolescentis* in IBD. The fecal level of *B. adolescentis* was decreased in the IBD patients compared with the normal people in our cohort and the GMrepo database. To further clarify the role of *B. adolescentis* in IBD, we induced chronic colitis with three cycles of dextran sulfate sodium (DSS). We found *B. adolescentis* gavage exhibited protective effects as evidenced by the significantly decreased diarrhea score, spleen weight, and increased colon length. Accordingly, the cumulative histological grading was decreased in the *B. adolescentis* administration group. In addition, tight junction protein and mucin family were enhanced after *B. adolescentis* treatment. Furthermore, distinct effects were found with decreased pro-inflammatory cytokines such as TNF-α, IL-6, IL-1β, IL-18, IL-22, IL-9 and increased anti-inflammatory cytokines IL-10, IL-4, IL-5. Importantly, the colon lamina propria in the *B. adolescentis* group consisted of more Treg and Th2 cells, which inhibited extreme gut inflammation. Additionally, 16srRNA sequencing showed an evident increase in the B:F ratio in the *B. adolescentis* group. In particular, *B. adolescentis* application inhibited the excessive growth of *Akkermansia* and *Escherichia-Shigella* in genus level. In conclusion, *B. adolescentis* refined the DSS-induced chronic colitis by stimulating protective Treg/Th2 response and gut microbiota remodeling. *B. adolescentis* regularly treatment might improve the therapeutic effects for inflammatory bowel disease.

## Introduction

Inflammatory bowel disease (IBD) is defined as a chronic intestinal inflammatory disease accompanied by hematochezia, abdominal pain, diarrhea, or indigestion.^1^ The incidence rate shows a yearly upward trend and it causes a major worldwide healthcare burden.^2^ There are some key pathways in the pathogenesis of IBD. Evidences suggest that genetic factors, defects in mucosal barrier function, innate and adaptive immunity, and altered microbial composition contribute to the initiation and the development of IBD.^3–5^

It is widely accepted that cytokines and their related immune cells play a key role in the occurrence of IBD. For example, cytokines determine the T cell differentiation of Th1, Th2, T regulatory (Tregs) and Th17 cells in IBD.^6^ Foxp3 Tregs are crucial in immune regulation via IL-10 secretion.^7^ Emerging evidence indicates that gut microbe might be an important factor in modulating immune responses affecting IBD progression. The study suggests that harmful bacteria antigens might activate antibacterial immune system which defend against pathogenic and finally induce immune hyperresponsiveness. Besides, microbial dysbiosis not only caused alterations in the epithelial mucosa, but also activated inflammation by regulating the activity of cytokines.^8^ Usually, IBD is accompanied by flora dysregulation. Thus, the composition and function of the gut ecosystem has a significant association with the occurrence of IBD. The study suggested that reduced gut microbiota diversity and loss of key species was necessary to maintain intestinal homeostasis.^9^

Currently, probiotics have been experimentally and mechanically investigated for their possible effectiveness in treating inflammatory bowel disease, immune therapy, cancer, and metabolic diseases.^10–13^ Numerous studies have found changes in IBD gut flora, and some researchers find that the declines mainly concentrate on *Bifidobacteria* and *Lactobacillus*.^14^ The genus *Bifidobacterium*, the predominant component of the intestinal flora, has a variety of probiotic functions in healthy humans. Several species of *Bifidobacterium* have shown anti-inflammatory activity^15,16^ In addition, *B. adolescentis* can protect mice from *Y. enterocolitica* infection with DSS-induced acute colitis.^17^ Moreover, *B. adolescentis* simultaneously attenuates HFD-induced obesity by improving the intestinal barrier function.^18^

The function of *B. adolescentis* on a host’s immune system as well as the detailed changes in the gut microbiome in chronic colitis are still not fully explored. In this study, we analyzed some known probiotics in online databases, then found *B. adolescentis* was one of the probiotics making a significant difference. Therefore, we investigated how the probiotic *B. adolescentis* improved the gut ecosystem in a murine chronic colitis model by regulating of Treg and Th2 cells and intestinal flora composition.

## Results

### The clinical relevance of B. adolescentis abundance with ulcerative colitis

**Figure 1.**
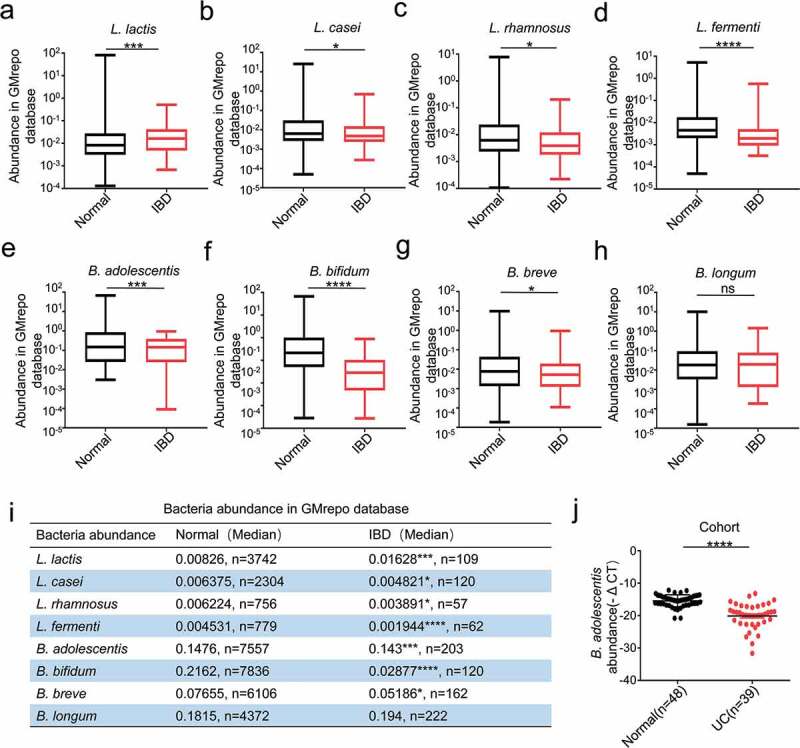
The clinical relevance of *B. adolescentis* abundance with ulcerative colitis

We first analyzed the *Lactobacillus* probiotic levels of *L. lactis, L. casei, L. rhamnosus* and *L. fermenti*, and the *Bifidobacteria* probiotic levels of *B. adolescentis, B. bifidum, B. breve* and *B. longum* in the stool of healthy individuals and IBD patients in the GMrepo database. We found reduced levels of *Lactobacillus* and *B. adolescentis* in the IBD patients when compared to the healthy people, but the levels of *L. lactis* and *B. longum* were increased in IBD patients (Figure 1a-h). The details of bacteria including exact sample numbers, medians and values were shown in the table (Figure 1i). Since our study focused on the function of *B. adolescentis* in the ulcerative colitis, so we next examined *B. adolescentis* abundance in the stool of 48 normal controls and 39 patients with ulcerative colitis from our hospital. As determined by quantitative PCR, *B. adolescentis* abundance (-ΔCT) was significantly lower in patients with UC than in the normal controls (Figure 1g). These results indicate that *B. adolescentis* is associated with the progression of ulcerative colitis.

(a-d) The fecal abundance of *L. lactis, L. casei, L. rhamnosus* and *L. fermenti* in the healthy and IBD patients in GMrepo database. (e-h) The fecal abundance of *B. adolescentis, B. bifidum, B. breve* and *B. longum* in the healthy and IBD patients in GMrepo database. (i) Details of the abundance of these bacteria including exact sample numbers, medians and values, the graph (in a-h) exactly matched with this table. (j) Bacteria genomic DNA was extracted from the stool of the healthy(n = 48) and IBD patients(n = 39), then the expression of *B. adolescentis* was tested by qPCR. *p < .05, ***p < .001, ****p < .0001, ns no significant (Mann-Whitney U test). IBD, inflammatory bowel disease; UC, ulcerative colitis.

### B. adolescentis ATCC15703 gavage ameliorated the DSS-induced chronic colitis

**Figure 2.**
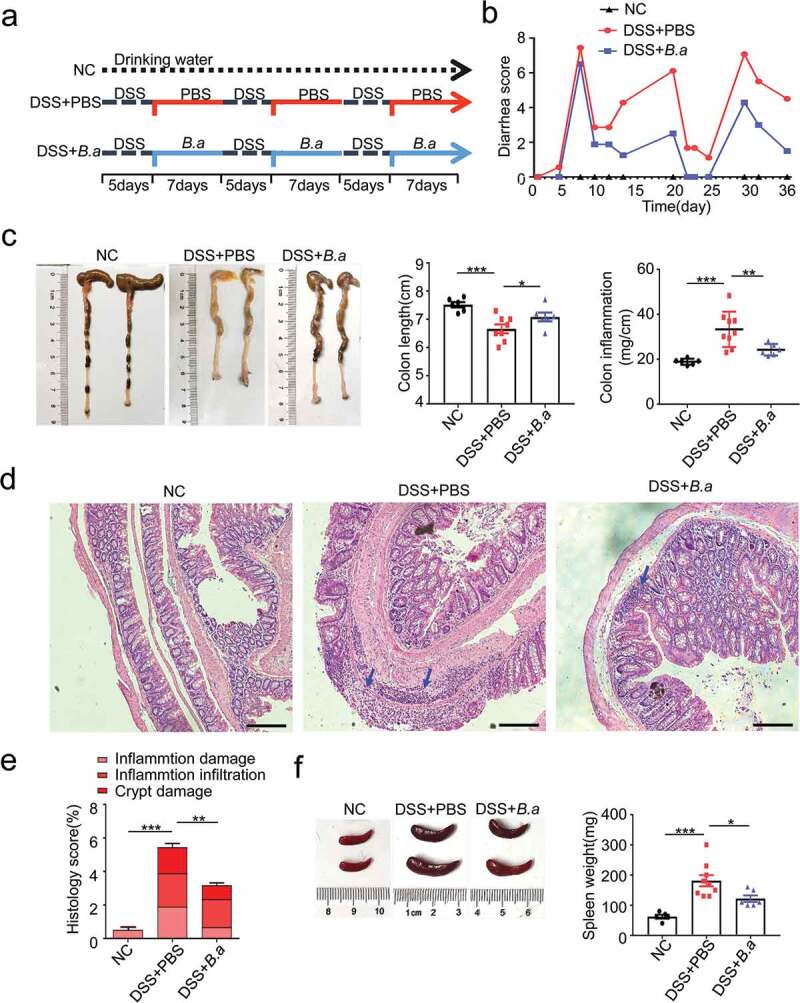
*B. adolescentis* ATCC15703 gavage ameliorated the DSS induced chronic colitis

To further clarify the role of *B. adolescentis* in IBD, we fed C57L/B6 mice with 3% DSS for 36 days plus *B. adolescentis* or PBS (Figure 2a). At first, we confirmed that *B. adolescentis* had the ability to colonize C57/B6 mice by testing the relative abundance of *B. adolescentis* in the stool and colon tissue compared *B. adolescentis* group and PBS group in the DSS model and Abx model (Supplementary Figure 1a-d). Then we found that mice gavage with *B. adolescentis* showed significantly lower diarrhea scores and a higher trend of body weight than mice gavage with PBS (Figure 2b and Supplementary Figure 1e). Besides, the *B. adolescentis* group showed longer colon lengths and decreased colon inflammation than the PBS group, which was confirmed by colon weight to colon length (Figure 2c). Moreover, histological examination of colon sections showed lower cumulative scores characterized by inflammation damage, inflammation infiltration and crypt damage in *B. adolescentis* fed mice compared with PBS fed mice (Figure 2d,e). Consistent with the pronounced chronic colitis seen on spleen weight (Figure 2f) was significantly lower in *B. adolescentis* mice compared to PBS mice. Taken together, these results suggest that *B. adolescentis* protects mice from the development of chronic colitis.

(a) Experimental animal model for chronic colitis. (b) Diarrhea score was recorded during the experiment according to rectal bleeding and diarrhea. (c) The entire colon was harvested on day 36. Colon length and colon inflammation were analyzed in the negative control mice and the PBS or *B. adolescentis* ATCC15703 gavage mice with DSS. (d) Representative pictures of H&E-stained (scale bars, 500 µm; blue arrows, inflammatory cells). (e) Cumulative scores including inflammation damage, inflammation infiltration and crypt damage were measured. (f) Spleen weight was measured. Data are expressed as mean ± SD, n = 6–9. *p < .05, **p < .01, ***p < .001 (ANOVA test). NC, negative control; DSS, dextran sulfate sodium; PBS, phosphate buffered saline; *B.a, Bifidobacterium adolescentis.*

### Administration of B. adolescentis ATCC15703 induces gut barrier reinforcement

**Figure 3.**
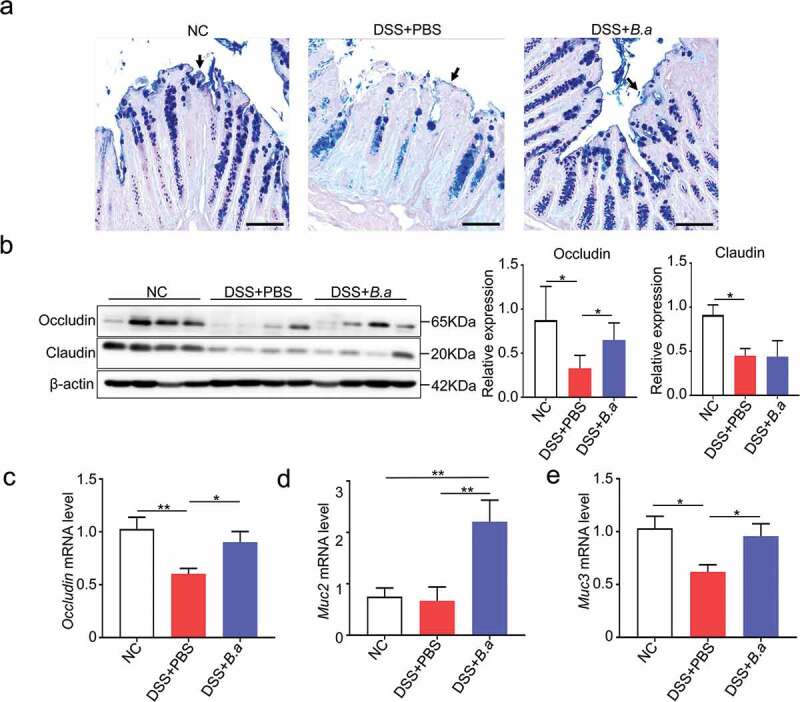
Administration of *B. adolescentis* ATCC15703 induces gut barrier reinforcement

To investigate the effect of *B. adolescentis* on the colon epithelium barrier in DSS induced colitis, we investigated the mucus layer thickness by Alcian blue & PAS staining. Mucus layer thickness has been thought to reflect the function of the gut barrier and protect epithelia from harmful factors.^19^ We found that mucus layer thickness was increased in *B. adolescentis* treated mice compared to PBS mice after DSS treatment (Figure 3a). Moreover, we found the protein level of occludin significantly increased in *B. adolescentis* treated mice compared to PBS mice after DSS treatment; meanwhile, claudin had no diffidence in these two groups (Figure 3b). Consistently, the mRNA expression level of gene *occludin* showed the same trend (Figure 3c). Occludin is an important transmembrane and intracellular tight junction.^5,20^ Mucin is a critical component that is made of the gut barrier,^21^ so we investigated the mucin family expression of muc2, muc3 in the experiment model. We found that the mRNA expression level of *muc2* and *muc3* increased in the *B. adolescentis* group compared to the PBS group (Figure 3d,e). These results indicate that *B. adolescentis* induced gut barrier reinforcement contributes to the restoration of chronic colitis.

(a) Representative pictures in Alcian blue & PAS stained (scale bars, 100 µm; arrows, mucus layer). (b) Western blot analysis of the tight junction related proteins like occludin and claudin in the colon tissue of the negative control mice and PBS or *B. adolescentis* ATCC15703 gavage mice with DSS. (c) The mRNA expression of the *occludin* gene in colon tissue. (d,e) The mRNA expression of gene *muc2* and *muc3*. Each experiment was repeated triplicate. Data are expressed as mean ± SD, n = 6–9. *p < .05, **p < .01 (ANOVA test or Kruskal-Wallis test). NC, negative control; DSS, dextran sulfate sodium; PBS, phosphate buffered saline; *B.a, Bifidobacterium adolescentis.*

### B. adolescentis ATCC15703 induces anti-inflammatory cytokines

**Figure 4.**
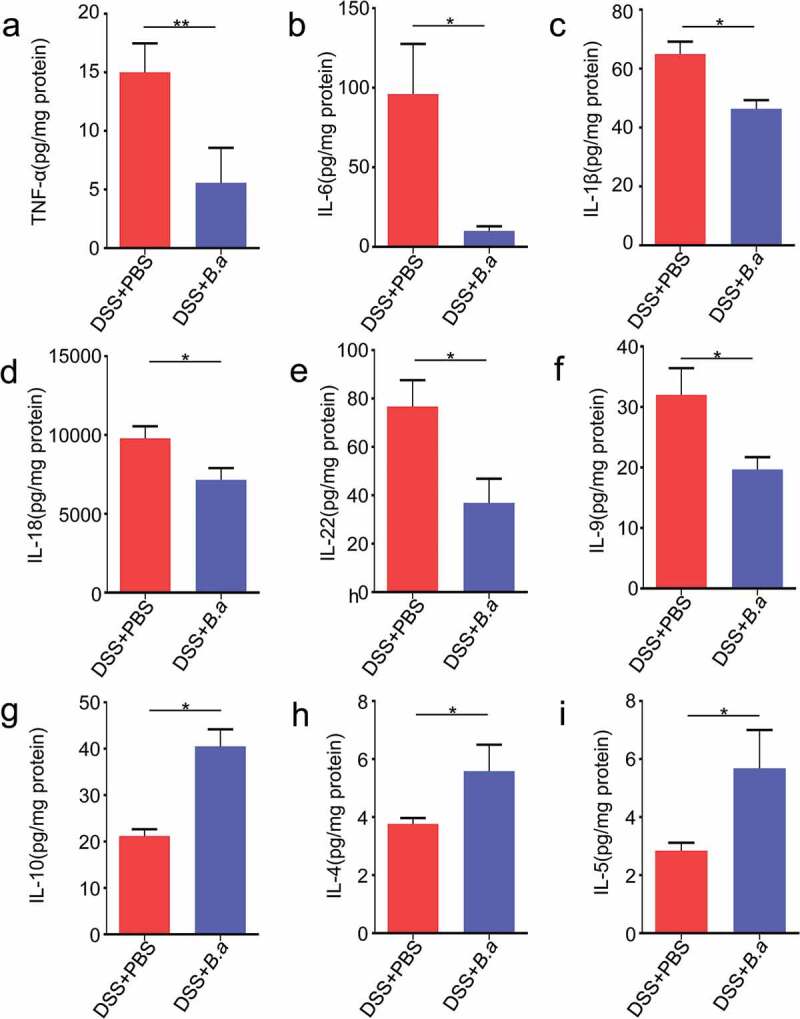
*B. adolescentis* ATCC15703 induces anti-inflammatory cytokines

To understand the reason why *B. adolescentis* could relieve chronic colitis, we assessed typical pro-inflammatory and anti-inflammatory cytokines, as macrophage infiltration and colon tissue damage were found by histological analysis in our study. To identify the cytokines secreted in the colon, the cytokine profiling of the colon homogenate supernatant from *B. adolescentis* mice and PBS mice were performed using the Th1/Th2/Th9/Th17/Th22/Treg Cytokine 17-Plex panel. As is shown in Figure 4, the Th17-type cytokines (Figure 4a) tumor necrosis factor alpha (TNF-α), (Figure 4b) IL-6, (Figure 4c) IL-1β were significantly decreased in colon homogenate supernatant from the *B. adolescentis* group compared to the supernatant from PBS group. Th1-type cytokine (Figure 4d) IL-18, Th22-type cytokines (Figure 4e) IL-22 and Th9-type cytokines (Figure 4f) IL-9 also decreased as well. Meanwhile, Treg-type cytokines (Figure 4g) IL-10, Th2-type cytokines (Figure 4h) IL-4 and (Figure 4i) IL-5 were increased in colon homogenate supernatant. Other cytokines such as IL-17A and IFN-γ showed no significant diffidence in these two groups (Supplementary Figure 1f). These results suggest that *B. adolescentis* may inhibit the secretion of pro-inflammatory cytokines and induce anti-inflammatory cytokines, so it can extenuate chronic colitis.

Concentrations of the Th17-type cytokines (a) Tumor necrosis factor alpha (TNF-α), (b) IL-6, (c) IL-1β; Th1-type cytokine (d) IL-18; Th22-type cytokines (e) IL-22; Th9-type cytokines (f) IL-9. Treg-type cytokine (g) IL-10, Th2-type cytokines (h) IL-4 and (i) IL-5 were performed by the Th1/Th2/Th9/Th17/Th22/Treg Cytokine 17-Plex Mouse ProcartaPlex™ Panel. Each experiment was repeated in triplicate. Data are expressed as mean ± SD, n = 6–9. *p < .05, **p < .01 (unpaired t test or Mann-Whitney U test). DSS, dextran sulfate sodium; PBS, phosphate buffered saline; *B.a, Bifidobacterium adolescentis.*

### B. adolescentis ATCC15703 triggered protective Treg and Th2 cell response in colon lamina propria

**Figure 5.**
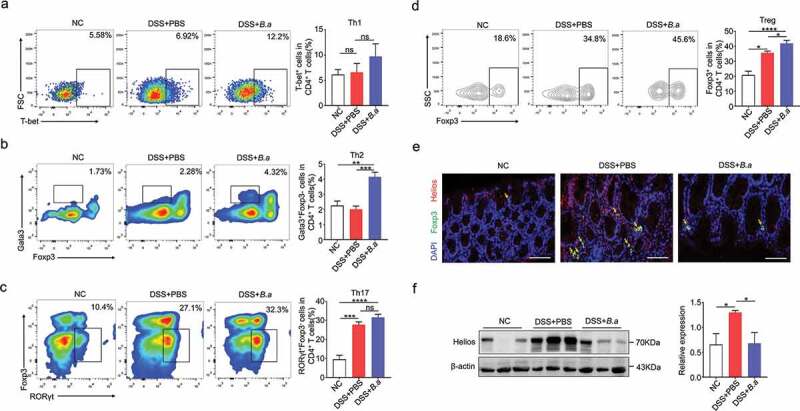
*B. adolescentis* ATCC15703 triggered protective Treg and Th2 cell response in colon lamina propria

Previous studies indicated that Treg suppressed colon inflammation by IL-10 secretion.^7^ On the other hand, Th2 cells mainly secreted IL-4 and its release in turn stimulated Th2 cell activation.^22^ As Treg-type cytokines IL-10, Th2-type cytokines IL-4 and IL-5 increased after *B. adolescentis* treatment in DSS model in our previous data, we subsequently attempted to identify the CD4^+^ T cell subtypes responsible for regulating colitis pathogenesis by *B. adolescentis*. We found that total T cells (CD4/CD3 cell: 20.3% vs.14%, *p* < .05) increased in *B. adolescentis* group fed with DSS (Supplementary Figure 2a). Moreover, compared to mice gavage with PBS, mice deal with *B. adolescentis* showed increased colon lamina propria Th2 cells (Th2/CD4 T cells: 4.32% vs.2.28%, *p* < .001) (Figure 5b) and Treg cells (Treg/CD4 T cells: 45.6% vs.34.8%, *p* < .05) (Figure 5d). In the while, we found other helper T cells Th1 and Th17 cells showed no significant diffidence in *B. adolescentis* group and PBS group fed with DSS (Figure 5a,c).

(a,b,c,d) Frequencies of Th1 cells (CD45^+^CD3^+^CD4^+^T-bet^+^)(a), Th2 (CD45^+^CD3^+^CD4^+^gata3^+^foxp3^−^) (b) Th17 (CD45^+^CD3^+^CD4^+^RORγt^+^foxp3^−^) (c) and Treg (CD45^+^CD3^+^CD4^+^foxp3^+^) (d) in the colon lamina propria of negative control mice and PBS or *B. adolescentis* gavage mice with DSS. (e) Representative pictures in immunofluorescence staining of foxp3 and helios staining in colon tissue of three groups (scale bars, 100 µm; yellow arrows, positive cells). (f) The protein level of helios in colon tissue. Data are expressed as mean ± SD, n = 4. *p < .05, **p < .01, ***p < .001, ****p < .0001 (ANOVA test). NC, negative control; DSS, dextran sulfate sodium; PBS, phosphate buffered saline; *B.a, Bifidobacterium adolescentis.*

Previous study reported that the expression of the transcription factor helios has proven to be a useful marker for the identification of stable nTregs, while nTregs could induce the suppression of autoimmune disease.^23^ To further investigate the exact Treg cells subset, we performed the immunofluorescence staining of foxp3 and helios, then we found that foxp3 and helios double positive cells decreased in the *B. adolescentis* group with DSS treatment when compared to the PBS group (Figure 5e). In addition, the protein level of helios in colon tissue significantly downregulated as well (Figure 5f). These data indicated that helios positive nTreg cells didn’t contribute to the rise of total Treg cells. In vitro, we cocultured isolated spleen cells and *B. adolescentis* to validate the result. As expected, the flow cytometry showed nTreg (CD3^+^ CD4^+^ Foxp3^+^ helios^+^ cells) decreased in the *B. adolescentis* group (Supplementary Figure 2b). These results suggest that *B. adolescentis* protects against the evolution of chronic colitis partly through the restoration of Treg and Th2 cells.

### Dextran sulfate sodium decreased microbial diversity and reshaped the microbial community

**Figure 6.**
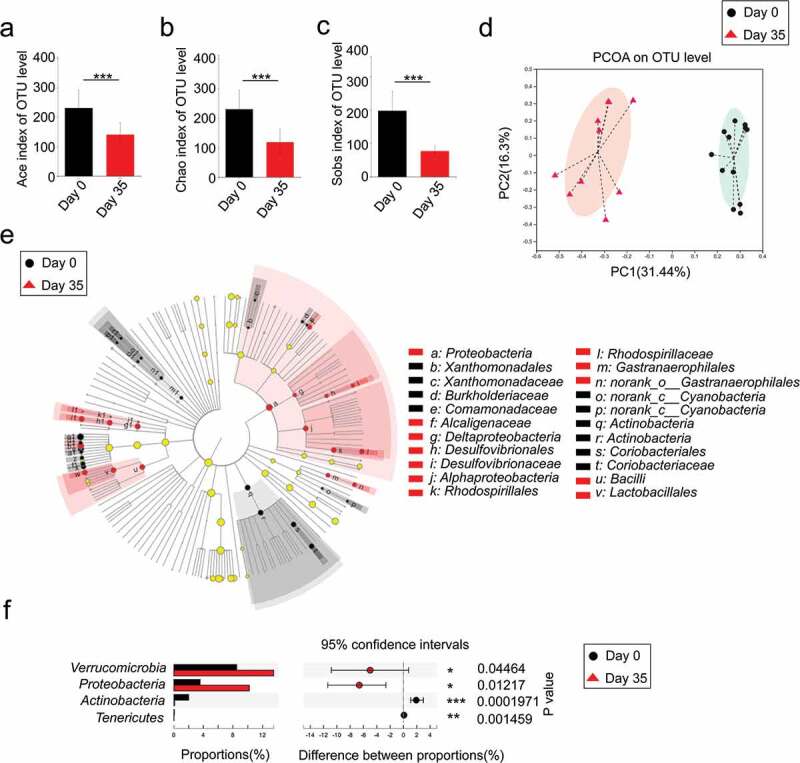
Dextran sulfate sodium decreased microbial diversity and reshaped the microbial community

A large number of studies have demonstrated that intestinal flora plays a critical role in the alleviation or development of IBD.^24^ Before investigating the influence of *B. adolescentis* microbial colitis, we first confirmed the microbial disorder and characteristics in dextran sulfate sodium-induced chronic colitis. To investigate the influence of DSS treatment on gut microbiota composition, we collected mice stool at the beginning of DSS model as a baseline, then collected stool on day 35 of DSS administration. Mice stool was sent for 16S rRNA sequencing. We found that stool bacteria commensal richness and diversity significantly decreased on day 35 of DSS administration compared to the primary day 0 time point, which was evident by ace, chao and sobs indexes (Figure 6a-c). Furthermore, we examined altered microbial construction by the principal coordinate analysis (PCoA) plot on OUT levels, which clearly showed the distance of sample groups (beta diversity). Moreover, we found PCoA plots were divided into two cohesive groups correspondence with the two time points of day 0 and day 35 (Figure 6d).

A microbiome analysis was conducted using stool samples collected from mice on day 0 and day 35 with DSS. (a-c) The α-diversity of the gut microbiome, determined by the Ace, Chao and Sobs diversity index of the primary day 0 group compared with the day 35 group with DSS. (d) The principal coordinate analysis (PCoA) plot shows the β-diversity of the gut microbiome. (e) Bacterial taxa identified as differentially abundant between groups according to linear discriminant analysis effect size (LEfSe). The bacterial taxa of the primary day 0 group were compared with those of the day 35 group with DSS ranging from phylum to family. Black indicates bacterial taxa whose abundance was higher in the day 0 group and red indicates bacterial taxa whose abundance was higher in the other group. (f) Significant phyla alterations in the primary day 0 group and the day 35 group with DSS. n = 9–11. *p < .05, ***p < .001, ****p < .0001 (unpaired t test or Wilcoxon rank-sum test).

To further characterize phenotypic changes in the taxonomic composition, we performed LEfSe analysis to identify differentially abundant biomarkers with biological consistency between day 0 and day 35 (Figure 6e). We found some enriched taxa were observed between the two groups at multiple phylogenetic levels. Then we compared relative taxa abundance at the phylum and family levels using Wilcoxon rank sum tests. There were significant changes in phyla at day 35 compared to the baseline day 0, including reduction in *Actinobacteria* and *Tenericutes* (*P* < .01) as well as an increased abundance of *Verrucomicrobia* and *Proteobacteria*(*P* < .05) (Figure 6f). These results indicate that three cycles of DSS induced chronic colitis along with microbial disorder.

### B. adolescentis ATCC15703 administration altered relative abundance of taxa at multiple levels in chronic colitis

**Figure 7.**
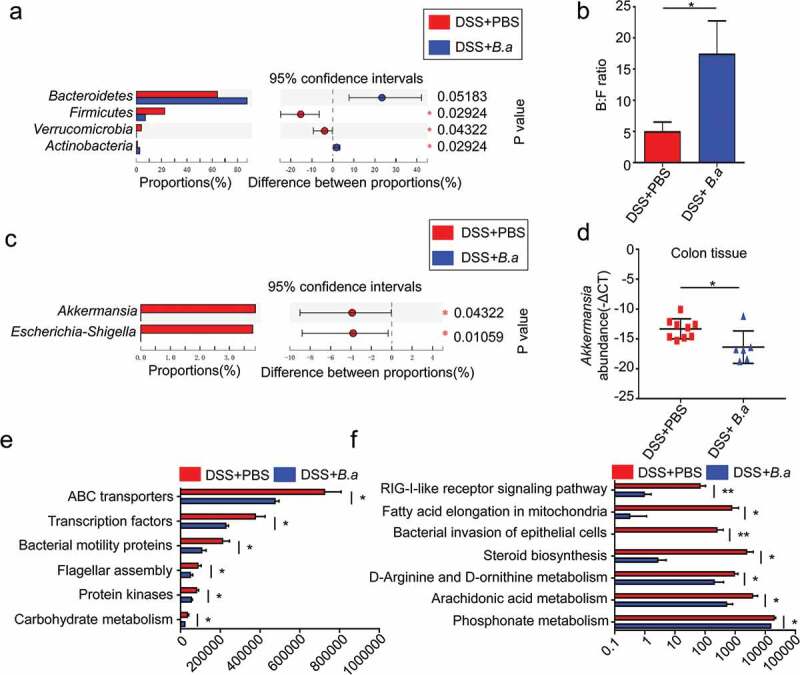
*B. adolescentis* ATCC15703 administration altered relative abundance of taxa at multiple levels in chronic colitis

To investigate the effect of *B. adolescentis* administration on gut bacteria remodeling of DSS-induced chronic colitis, mice stool was collected in the final day for 16S rRNA sequencing. After continuous administration of *B. adolescentis, Firmicutes* and *Verrucomicrobia* abundance substantially decreased and the *Actinobacteria* proportion increased (3% in *B.a* group, 1% in control group, *P* < .05), which was due to the treatment (Figure 7a). Similarly, there was an increase in the *Bacteroidetes: Firmicutes* (B: F) ratio from 5 to 17 after *B. adolescentis* treatment (Figure 7b). We also found that bacteria treated mice harbored a distinctively lower genus abundance of *Akkermansia* and *Escherichia-shigella* compared to that of the untreated mice (Figure 7c). In addition, we further examined the *Akkermansia* level in colon tissue on *B. adolescentis* treated and untreated mice. We found the *Akkermansia* level decreased about fourfold changes in *B. adolescentis* group tissue when compared to the control group (Figure 7d). The abundance change of *Akkermansia* indicates that the influence of *B. adolescentis* on the remission of colitis through restricting *Akkermansia* excessive reproduction.

A microbiome analysis was conducted using colorectal stool samples collected from 6 to 9 mice per group at day 36 in the PBS group and the *B.a* group with DSS. (a) Significant phyla alterations in the PBS group and the *B.a* group with DSS. (b) *Bacteroidetes: Firmicutes* (B: F) ratios in different groups. (c) Significant genus alterations in the PBS group and the *B.a* group with DSS. (d) Bacteria genomic DNA was extracted from mice colon tissue from the PBS group and the *B.a* group with DSS, genus *Akkermansia* level was tested by qPCR. (e,f) Pathways that are predicted to show significant different abundances between the PBS group and the *B.a* group with DSS according to the Kyoto Encyclopedia of Genes and Genome (KEGG) pathway analysis. n = 6–9. *p < .05, **p < .01(unpaired t test or Wilcoxon rank-sum test). PBS, phosphate buffered saline; *B.a, Bifidobacterium adolescentis.*

To further elucidate the effect of *B. adolescentis* on gut bacteria function, we used the Kyoto Encyclopedia of Genes and Genome (KEGG) pathway analyses for function prediction. We found 13 pathways were significantly different between *B. adolescentis* and the control group. Some microbiota characteristic pathways were predicted to be less active in the *B. adolescentis* group, such as bacteria motility protein and bacteria invasion signaling pathways. Many important metabolic pathways were modulated to be more active in the control group compared to the *B. adolescentis* group, such as fatty acid elongation in mitochondria and steroid biosynthesis (Figure 7e,f).

## Discussion

In this study, we demonstrated that the *B. adolescentis* ATCC15703 strain regulates the immune response and improves the symptoms of IBD via the gut microbiota. Genus *Lactobacillus, Bifidobacterium* and *Lactococcus* have been developed as probiotics and contribute to modulating intestinal inflammation. Many databases outline human gut metagenomic data covering many disease phenotypes. Among them, we searched a new database called GMrepo. The GMrepo is a curated and annotated human gut metagenomic data repository for microbiota. In the GMrepo database, we found *B. adolescentis* decreased in the IBD patients compared with the normal people (Figure 1). Accumulating evidence has indicated this bacterium has antiviral properties^25^ and ameliorates metabolic system inflammation and is considered a promising probiotic.^26^ However, few studies have focused on its microbial alteration and immune modulation, and whether *B. adolescentis* also has a favorable role in DSS related chronic colitis. Consistent with our assumption, we found oral of *B. adolescentis* relieved the symptoms of chronic colitis (Figure 2).

In general, DSS can induce colon epithelial damage^27^ and *B. adolescentis* acts as a short-chain fatty acid-producing bacteria.^16^ Its extra supplementation may affect intestinal epithelial healing. Thus, we confirmed whether a weakened intestinal epithelial barrier could be restored by *B. adolescentis*. We found that *B. adolescentis* reinforced the epithelial barrier by increasing the colon’s mucus layer thickness and junction proteins (Figure 3). Mucus layer thickness has been thought to reflect the function of the gut barrier and protect epithelia from harmful factors.^19^ Occludin is an important transmembrane and intracellular tight junction.^5,20^ Similarly, the mucin family is a critical component of the gut barrier.^28^ Alterations in tight junction protein expression and distribution have been considered key factors in the onset of colonic inflammation and UC.

In addition, previous histological analysis showed that *B. adolescentis* treatment recovered mucosal and crypt structures by lessening the infiltration of inflammatory cells into the mucosa and sub-mucosa. Th1, Th2, and Th17 cells are involved in the pathogenesis of DSS-induced colitis.^29^ A previous study showed that targeted impairment of Th17 cell function could treat CD.^30^ Besides, transient depletion of Treg cells increased the severity of DSS-induced colitis.^31^ A previous study demonstrated that *B. adolescentis* specifically activated Th17 cells, but this effect didn’t induce colon inflammation.^32^ In our experiment, supplement of *B. adolescentis* decreased the pro-inflammatory cytokines but increased the anti-inflammatory cytokines of IL-10 and IL-4. While non-T cell populations, such as macrophage and dendritic cells always release pro-inflammatory cytokines of TNF-α, IL-6 or IL1β, but these cytokines were decreased after *B. adolescentis* treatment in our study (Figure 4). In addition, we further test the population of macrophages and dendritic cells in the antibiotics (Abx) model. Then we found that *B. adolescentis* treatment didn’t influence these two populations in Abx model by flow cytometry (Supplementary Figure 3). The cytokine of IL-22 decreased after *B. adolescentis* treatment, but it was reported that IL-22 from innate lymphoid cells 3 (ILC3) protects intestinal maintenance,^33^ which cannot be well understood in our study and needs further exploration. Treg cell and Th2 cell populations in colon lamina propria increased after DSS treatment compared with the normal control in our study. This is consistent with the previous study that DSS has the characteristic of inducing the proportion of T helper cells and Treg cells.^34^ Meanwhile, *B. adolescentis* administration increased Treg cell and Th2 cell subsets compared to the PBS group with DSS (Figure 5). It is well known that Treg and Th2 cells have the ability of limiting inflammation and weakening the role of pro-inflammatory factors.^35^ Overall, *B. adolescentis* may alter the mucosal immune response accompanying the secretion of anti-inflammatory cytokines and triggering Th2 and Treg cell response. However, whether *B. adolescentis* is sufficient to induce Treg and Th2 cells in germ-free or gnotobiota mice need further exploration.

A large number of studies have demonstrated that intestinal flora plays a critical role in the alleviation or development of IBD and that inflammation via IBD can in turn drive the loss of microbiota diversity.^24,36^ There was an increase in the *Bacteroidetes: Firmicutes* (B: F) ratio in the *B. adolescentis* group compared with the PBS group. (Figure 7). B:F ratio has been reported to be associated with aging and metabolic syndrome.^37,38^ In our study, we found a lower genus abundance of *Akkermansia* and *Escherichia-shigella* in the *B. adolescentis* group. Escherichia-Shigella is an adherent-invasive bacterium, which increased in the UC group and aggravated the development of colitis.^39,40^ Now some argue whether or not *Akkermansia* acts as a probiotic. Recent studies have reported that *Akkermansia* repaired epithelial damage, strengthened the mucosal barrier and protected from both acute and chronic colitis.^41,42^ In contrast, a study pointed out that it acted as a pathogen to promote colitis in a genetically susceptible host.^43^ Predicted functions on the KEGG pathway level showed bacteria motility protein and bacteria invasion signaling pathways were less active in the *B. adolescentis* group. The bacteria ability of motility and invasion is connected with the bacteria’s virulence and pathogenicity to host.^39^ Previous studies demonstrated that short-term application of probiotics could affect the abundance and composition of specific intestinal flora.^44^ However, associated taxa at the species level in our study were not investigated due to the sequence length. The specific functions of each microbial taxa in the gut microbiota need further evaluation.

In conclusion, *B. adolescentis* refined the DSS-induced chronic colitis by stimulating protective Th2/Treg response and gut microbiota remodeling. Our study suggests *B. adolescentis* may guide therapeutic strategies to control IBD by manipulating host immune response and the microbiota. Regular *B. adolescentis* treatment might improve therapeutic effect for inflammatory bowel disease.

## Materials and methods

### GMrepo database analysis

Phenotypes associated relative microbial abundance information at the species level was acquired from the GMrepo database. The GMrepo is a curated and annotated human gut metagenomic data repository for microbiota.^45^ Bacteria relative abundance were calculated at the species level for each sample, with a total abundance value of 100%. The detailed information can be searched in the web of gmrepo.humangut.info/help. Using GMrepo RESTful APIs for R (version 3.6.1 https://www.r-project.org) and RStudio (version 1.1.442 https://www.rstudio.com) software, we obtained some typical microbiota relative abundances in the stool samples of the healthy and IBD patients from GMrepo. Firstly, we assessed the data quality by consulting the description of the samples and supplementary data of related publications. Then, the relative abundances of interested microbiota for the healthy and IBD patients were extracted. *L. lactis, L. casei, L. rhamnosus, L. fermenti* and *B. adolescentis, B. bifidum, B. breve, B. longum* relative abundances were analyzed.

### Human stool sample collection

Stool samples were obtained from 39 patients with ulcerative colitis and 48 normal people for health examination from Sir Run Run Shaw Hospital, School of Medicine, Zhejiang University. Patients with ulcerative colitis were diagnosed by colonoscopy and pathology. Patients with ulcerative colitis were at the active stage without immunity therapy or drug treatment. Control individuals had no history of diarrhea or use of antibiotics or probiotics in the past month. All participants provided written informed consent before collection and the Clinical Research Ethics Committee of the Sir Run Shaw Hospital, School of Medicine at Zhejiang University approved the study protocol. Fresh stool samples were collected from the UC patients or healthy controls and were immediately frozen and stored at −80°C.

### DNA extraction and bacteria quantification

Bacteria DNA from human fecal samples or mice stool were extracted using QIAGEN stool kits (QIAGEN, Germany) and bacteria genomic DNA from mice colon tissue were extracted using QIAGEN DNA mini kits (QIAGEN, Germany). Quantitative real-time PCR was performed in ROCHE LightCycler®480 System (Rotor gene 6000 Software, Sydney, Australia). Each reaction was tested in triplicate including SYBR Premix Ex Taq (Takara, Japan), primers and template DNA. Relative abundance was calculated by -ΔCt method. Universal Eubacteria 16s was used as internal reference gene. The primer sets used were:

*B. adolescentis: Forward: 5ʹ-CTCCGCCGCTGATCCGGAAGTCG-3ʹ,*

*Reverse: 5ʹ-AACCAACTCGGCGATGTGGACGACA-3ʹ;*

*Akkermansia: Forward: 5ʹ- AGAGGTCTCAAGCGTTGTTCGGAA-3ʹ,*

*Reverse: 5ʹ- TTTCGCTCCCCTGGCCTTCGTGC-3ʹ;*

*universal Eubacteria 16s: Forward: 5ʹ-CGGCAACGAGCGCAACCC-3ʹ,*

*Reverse: 5ʹ-CCATTGTAGCACGTGTGTAGCC-3*.^46^

### Preparation of B. adolescentis

*B. adolescentis* ATCC15703 was purchased from American type culture collection (ATCC, USA). V4 of 16S ribosomal RNA sequencing was performed to confirm bacterial strain at the species level. The bacteria were cultured in anaerobic modified Reinforced Clostridium Medium (BD Difco, Sparks, MD, USA) under an atmosphere of 10% H2, 10% CO2, and 80% N2 (AW500SG anaerobic workstations; ELECTROTEK, England) for 24 h. The cultures were centrifuged at 3000 rpm for 5 min at 4°C and then washed twice with sterile anaerobic PBS, then resuspended at a final concentration of 1 × 10^9^ CFU/300 µl under strictly anaerobic conditions. For analysis of the viability of *B. adolescentis*, the suspension was inoculated on RCM containing 1% agarose and then incubated for at least 2 days at 37°C in the anaerobic incubator.

### Chronic colitis model and treatments

Male age-matched C57L/B6 mice (6 ~ 8 weeks of age) were purchased from Shanghai SLAC Laboratory Animal, China. All animals were kept under specific pathogen-free conditions. Before bacterial intragastric administration, mice were fed with 2 mg/ml streptomycin in the drinking water for 3 days to ensure the consistency of regular microbiota, then they were randomly assigned to three groups. Five days of 3% DSS administration followed by 7 days of normal water, this process repeated for three cycles. Bacteria treatment was done after every DSS application. The bacteria were applied by oral gavage daily except when DSS was applied. Group *B.a* (DSS+*B.a*) mice were administrated 1 × 10^9^ CFU of *B. adolescentis* suspended in 300 µl sterile anaerobic PBS by oral gavage, while mice in the group control (DSS+PBS) were given an equivalent volume of sterile anaerobic PBS instead. The last group, normal, was given regular water. The treatment was continued for 36 days. Body weight was recorded weekly. Colon and spleen tissues were then harvested after mice were fasted at the end of the experiment. Colon tissue was photographed and length measured, while spleen tissue was photographed and weighed.

Diarrhea scores were recorded daily, rectal bleeding, and diarrhea were scored. Rectal bleeding: 0 = no bleeding, 2 = slight bleeding, and 4 = gross bleeding; diarrhea: 0 = well-formed stools, 2 = soft and pasty stools, and 4 = watery stools.^47^

Colons were opened longitudinally and weighted after removing the stool inside (excluding the cecum). Assessment of colon inflammation was performed by the colon weight (mg) normalized by its length (cm). All animal studies were performed in accordance with the guidelines of the Institutional Animal Use and the Animal Experimentation Ethics Committee at Zhejiang University. All mice were maintained in ventilated cages with 12-h light/dark cycles, enriched water and ad libitum feeding.

### Abx model and treatment

Male age-matched C57L/B6 mice (6 to 8 weeks of age) were purchased for building antibiotic mice. As previous studies reported,^48^ mice oral antibiotics (Abx) of 1 g/L ampicillin (C1613, sigma), 0.35 g/L vancomycin (PHR1732, sigma), 1 g/L metronidazole (M1547, sigma) and 1 g/L neomycin (N6386, sigma) for 2 weeks to abolish most bacteria. Then they were randomly assigned to two groups, the *B.a* group were administrated 1 × 10^9^ CFU of *B. adolescentis* suspended in 300 µl sterile anaerobic PBS by oral gavage per day, while mice in the PBS group were given an equivalent volume of sterile anaerobic PBS instead. Bacteria gavage lasted for 2 weeks until the mice were harvested.

### Assessment of colon inflammation

Colon tissue was settled in a cycle, then fixed overnight with 10% formalin at room temperature and then embedded in paraffin. Sections were stained with hematoxylin and eosin (H&E) for pathological analysis. Two independent investigators who were blinded to the treatment evaluated the slides. A 0–4 point scale was used to describe the severity of inflammation (0 = none, 1 = mild, 2 = moderate, and 3 = severe), the level of inflammation involvement (0 = none, 1 = mucosa, 2 = mucosa and submucosa and 3 = transmural) and the extent of epithelial/crypt damage (0 = none, 1 = basal 1/3, 2 = basal 2/3, 3 = crypt loss, 4 = crypt and surface epithelial destruction). Each parameter was calculated and summed to obtain the overall score.^47^

### Immunofluorescence staining

For immunofluorescence, sections of paraffin-embedded tissue were stained by foxp3 antibody (Servicebio, China), helios antibody (#89270, CST) and DAPI (solarbio). Two investigators who were blinded to the treatment evaluated the slides independently.

### Western blot analysis

Colon tissue was weighed and homogenized in RIPA extraction buffer (Solarbio, China). The homogenate was centrifuged at 4°C for 15 min at 15,000 g, then the supernatant was collected. The protein concentration was quantified with BCA protein assay kits (Solarbio, China) according to the manufacturer’s instructions. Proteins were separated by 10% SDS polyacrylamide gel, and then transferred onto PVDF membranes. The membranes were blocked with 5% skimmed milk for 1 h and then immunoblotted with primary antibodies against occuludin (ab167161, Abcam), claudin (36–4800, Thermo Fisher Scientific), helios (#89270, CST) at 4°C overnight. Membranes were then incubated with second antibodies labeled with HRP at room temperature for 1 h and bands were visualized using an ECL kits (Fdbio science, China). β-actin was used as a reference gene.

### RNA extraction and quantitative real-time PCR

RNA was extracted from the colon tissues of mice by using TRIzol reagent (Takara, Japan). Total RNA was reverse transcribed using PrimeScript™ RT reagent Kit (Takara, Japan). qRT-PCR was performed using SYBR Premix Ex Taq (Takara, Japan) in the Light Cycler®480 Real-Time PCR System (Roche) using cDNA. The mRNA expressions of mouse genes were analyzed with the specific primers listed below. The relative mRNA expression was calculated using the comparative cycle method (2^−ΔΔCt^). GAPDH served as internal reference genes.

*Occludin: Forward: 5ʹ-TTGAAAGTCCACCTCCTTACAGA-3ʹ,*

*Reverse: 5ʹ-CCGGATAAAAAGAGTACGCTGG-3ʹ;*

*Muc2: Forward: 5ʹ-ATGCCCACCTCCTCAAAGAC-3ʹ,*

*Reverse: 5ʹ-GTAGTTTCCGTTGGAACAGTGAA-3ʹ;*

*Muc3: Forward: 5ʹ-GCCGTGAATTGTATGAACGGA-3ʹ,*

*Reverse: 5ʹ-CGCAGTTGACCACGTTGACTA-3ʹ*;

### Cytokine profiling assay

Firstly, we selected all colon sections near anus. As reported in the previous study,^49^ colons were cut into small parts and PBS with protease inhibitor (Solarbio, China) was added following thawing and weighing, then homogenization was conducted on ice followed by centrifugation (5 min at 13,000 rpm). The supernatant was then stored at −80°C until analysis. Next, the supernatant was normalized using BCA protein assay kits (Solarbio, China) according to the manufacturer’s instructions. TNF-α, IL-6, IL-1β, IL-18, IL-22, IL-9, IL-10, IL-4, IL-5, IFN-γ and IL-17A were measured for cytokine profiling assay using the Th1/Th2/Th9/Th17/Th22/Treg Cytokine 17-Plex Mouse ProcartaPlex™ Panel (EPX170-26087-901, Thermo Fisher Scientific). Then the 17 mouse cytokine levels were determined using Bio-Plex 200 System (Bio-Rad Laboratories).

### Isolation of colon lamina propria cells

As previous studies reported,^50^ to isolate the colon lamina propria cells, colon tissue was cut into small pieces and incubated in epithelial cell solution including HBSS Ca/Mg-Free buffer (Solarbio, China) supplemented with 2% fetal bovine serum, 1 mM DTT and 5 mM EDTA at 37°C shaker for 30 min. The remaining colon pieces were cut into 1 mm pieces and further incubated in enzymatic digestion solution including HBSS buffer supplemented with 400 IU/mL Type Ⅳ collagenase (Sangon, China) and 10 mg/mL DNase I (Sangon, China) for 1 h at 37°C shaker. After complete digestion, the cell suspension was passed through a 200-mesh filter, and then cell suspension was centrifuged at 300 g for 5 min. The isolated colon lamina propria cells were collected for further analysis.

### Flow cytometry analysis

Colon lamina propria cells were subsequently counted, and the surface of the cells were stained for 30 min at room temperature using Fixable viability stain 510 (564406, BD Biosciences) for live cell staining, Alexa Fluor 700 anti-mouse CD45 (560510, BD Biosciences), PECP-CY5.5 anti-mouse CD3 (551163, BD Biosciences) and BV605 anti-mouse CD4 (563151, BD Biosciences) for cell surface staining. Next, cells were permeabilized with fixation/permeabilization buffer (eBioscience, San Diego, CA, USA) and cells were stained for 40 min at room temperature using PE-CY7 anti-mouse foxp3(560408, BD Biosciences), BV421 anti-mouse gata3 (563349, BD Biosciences), AF647 anti-mouse T-bet (561267, BD Biosciences), PE anti-mouse RORγt (IC6006P-025, R&D), APC anti-mouse helios (137222, Biolegend) for intracellular staining. Samples were analyzed using Flow Cytometer (BD Biosciences). Subsequent analysis was performed with FlowJo software (Tree Star Inc., San Carlos, CA). CD4 T cells were identified as CD45^+^CD3^+^CD4^+^; Treg cells were identified as CD45^+^CD3^+^CD4^+^foxp3^+^; Th2 cells were identified as CD45^+^CD3^+^CD4^+^gata3^+^foxp3^−^; Th1 cells were identified as CD45^+^CD3^+^CD4^+^T-bet^+^; Th17 cells were identified as CD45^+^CD3^+^CD4^+^RORγt ^+^foxp3^−^. nTreg were identified as CD45^+^CD3^+^CD4^+^Foxp3^+^helios^+^.

### Cell culture and treatment

Spleens were separated from C57L/B6 mice, followed by dissociating the spleens in red blood cell lysis buffer (Solarbio, China). Then spleen cells were cultured in RPMI-1640 medium (Genom, China) supplemented with 10% fetal bovine serum (FBS) and 1% penicillin and streptomycin. Cells were cultured at 37°C in humidified 5% CO2 atmosphere. Next, cells were cocultured with *B. adolescentis* for 48 h (MOI = 1:100) in antibiotic-free medium. Finally, the percentage of nTreg cells were analyzed by flow cytometry.

### DNA isolation, PCR amplification and sequencing

Bacteria genomic DNA was extracted from the mice colon contents with TIANamp Stool DNA Kits (TIANGEN BIOTECH, cat. #DP328-02, Beijing, China) according to the manufacturer’s instructions. DNA concentration and purification were measured with a Nanodrop 2000 UV–vis spectrophotometer (Thermo Scientific, Wilmington, USA) and DNA quality was checked by 1% agarose gel electrophoresis. The V3-V4 hypervariable regions of 16S ribosomal RNA genes were amplified using barcoded primers 338 F and 806 R by thermocycler PCR system (GeneAmp 9700, ABI, USA). The PCR amplicons were purified by AxyPrep DNA Gel Extraction Kit (Axygen Biosciences, Union City, CA, USA), and quantified by QuantiFluor™ -ST (Promega, USA). Purified amplicons were sequenced on an Illumina MiSeq platform (Illumina, San Diego, USA) according to the standard protocols by Majorbio BioPharm Technology Co. Ltd. (Shanghai, China).

### Taxonomic analyses of the gut microbiota

Raw fastq files were demultiplexed and quality filtered by Trimmomatic and merged by FLASH (Fast Length Adjustment of Short Reads to Improve Genome Assemblies). Samples were identified by barcodes and primers, then sequences were de-replicated and discarded. Operational Taxonomic Units (OTUs) were clustered with 97% similarity cut off using UPARSE (version 7.1 http://drive5.com/uparse/). The relative abundances of the microbial taxa (genus to kingdom) were generated from nonrarefied operational taxonomic unit tables. We used Ace, Chao and Sobs diversity index to measure species richness (α-diversity) of the gut microbiome. β-diversity of the gut microbiome was calculated using the UniFrac distance between samples and visualized using the principal coordinate analysis (PCoA) (http://www.majorbio.com/).

### Functional predictions

The 16S rRNA functional prediction was performed by normalizing the OTU abundance table through PICRUSt (Phylogenetic Investigation of Communities by Reconstruction of Unobserved States).^51^ Then OTUs were analyzed into Clusters of Orthologous Groups (COG)^52^ and Kyoto Encyclopedia of Genes and Genome (KEGG) orthology (KO).^53^ According to the COG database, the descriptive and functional information of each COG were parsed from the eggNOG database to obtain a functional abundance spectrum. KO, Pathway and Enzyme (EC) information was obtained in the KEGG pathway database while the abundance of each functional category was calculated according to OTU abundance.

### Statistical analysis

Data were expressed as mean ± standard deviation (SD) and were analyzed by unpaired Student’s t test or Mann–Whitney U test or one-way ANOVA test or Kruskal–Wallis test. Stool alterations in different experiment groups were compared by Wilcoxon rank sum tests. Mothur (v.1.30.1) was used to calculate indices of alpha diversity (Ace, Chao and Sobs index). Differences in alpha diversity and predicted pathway abundances were analyzed with the Wilcoxon rank sum test. A *p* value threshold of 0.05 (Wilcoxon rank sum test) and an effect size threshold of 2 were used for all bacterial taxa. A *P* value < .05 was considered statistically significant. Statistical analyses were performed using GraphPad Prism 5.04 software (GraphPad Software, Inc., La Jolla, CA, USA) and SPSS 19.0 for Windows (SPSS Inc., Chicago, IL, USA).

## Supplementary Material

Supplemental Material
