# Supplementary material for: B. adolescentis ameliorates chronic colitis by regulating Treg/Th2 response and gut microbiota remodeling
Source: Gut Microbes. 2021 Feb 9;13(1):1826746. doi: 10.1080/19490976.2020.1826746 (PMC7889144; doi:10.1080/19490976.2020.1826746)
Supplement: Supplemental Material [file KGMI_A_1826746_SM2661.zip › Supplementary information/GUT MICROBES-Supplenmentary figure legend.docx]

**Supplementary Figure 1.** ***B. adolescentis* had the ability of colonization in C57/B6 mice.** (a,b)The relative abundance of *B. adolescentis* in the stools and tissues of *B. adolescentis* or PBS treatment mice with DSS. (c,d) The relative abundance of *B. adolescentis* in the stools and tissues of *B. adolescentis* or PBS treatment mice with Abx treatment.(e) The body weight changes in group *B. adolescentis* or PBS with DSS. (f) Concentrations of IL-17A and IFN-γ were performed by the Th1/Th2/Th9/Th17/Th22/Treg Cytokine 17-Plex Mouse ProcartaPlex™ Panel. Data are expressed as mean ± SD or SEM, n = 6–9. *p <0.05, ***p <0.001, ns no diffidence (unpaired t test or Mann-Whitney U test). DSS, dextran sulfate sodium; PBS, phosphate buffered saline; *B.a*, *Bifidobacterium adolescentis*.

**Supplementary Figure 2.** ***B. adolescentis* induced nTreg response *in vitro*.** (a) Frequencies of total CD4 T cells (CD45^+^CD3^+^CD4^+^) in the colon lamina propria of negative control mice and PBS or *B. adolescentis* gavage mice with DSS. (b) The isolated spleen cells were cocultured with *B. adolescentis* for 48 h, then the level of nTreg (CD45^+^CD3^+^CD4^+^Foxp3^+^helios^+^ cells) was tested by flow cytometry. Data are expressed as mean ± SD, n = 5. *p <0.05, ns no diffidence (ANOVA test). DSS, dextran sulfate sodium; PBS, phosphate buffered saline; *B.a*, *Bifidobacterium adolescentis*.

**Supplementary Figure 3.** ***B. adolescentis* didn’t influence macrophage and** **dendritic cell in Abx mice.** (a) Frequencies of macrophage (CD45^+^F4/80^+^CD11b^+^) in the colon lamina propria of PBS or *B. adolescentis* gavage mice with Abx treatment. (b) Frequencies of dendritic cell (CD45^+^CD11b^+^MHCⅡ^+^) in the colon lamina propria of PBS or *B. adolescentis* gavage mice with Abx treatment. Data are expressed as mean ± SD, n = 6. ns no diffidence (unpaired t test). PBS, phosphate buffered saline; *B.a*, *Bifidobacterium adolescentis*.
