# Supplementary figures and images for: B. adolescentis ameliorates chronic colitis by regulating Treg/Th2 response and gut microbiota remodeling
Source: Gut Microbes. 2021 Feb 9;13(1):1826746. doi: 10.1080/19490976.2020.1826746 (PMC7889144; doi:10.1080/19490976.2020.1826746)

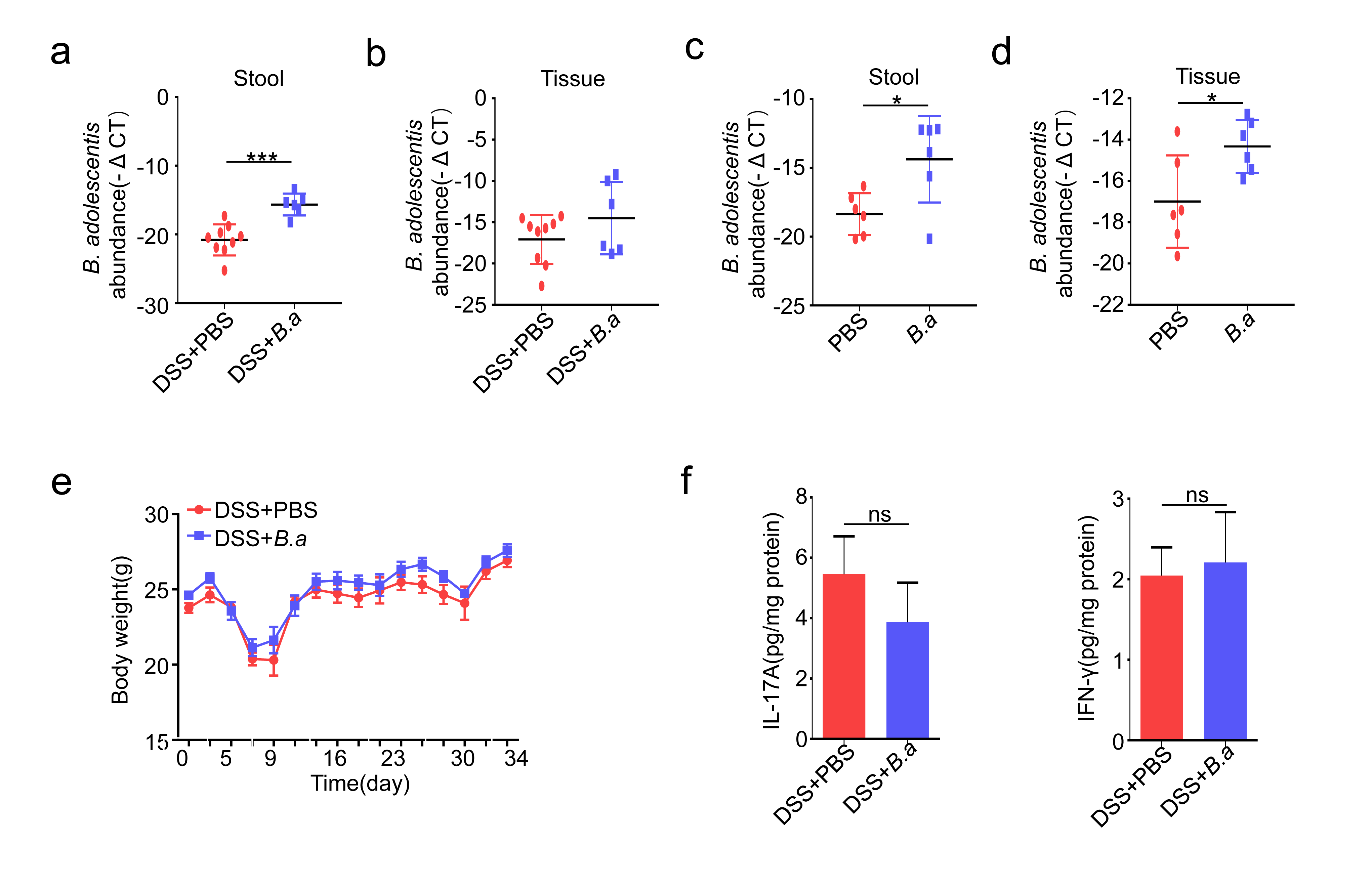

Supplement: Supplemental Material [file KGMI_A_1826746_SM2661.zip › Supplementary information/Supplementary Figure 1.tif]

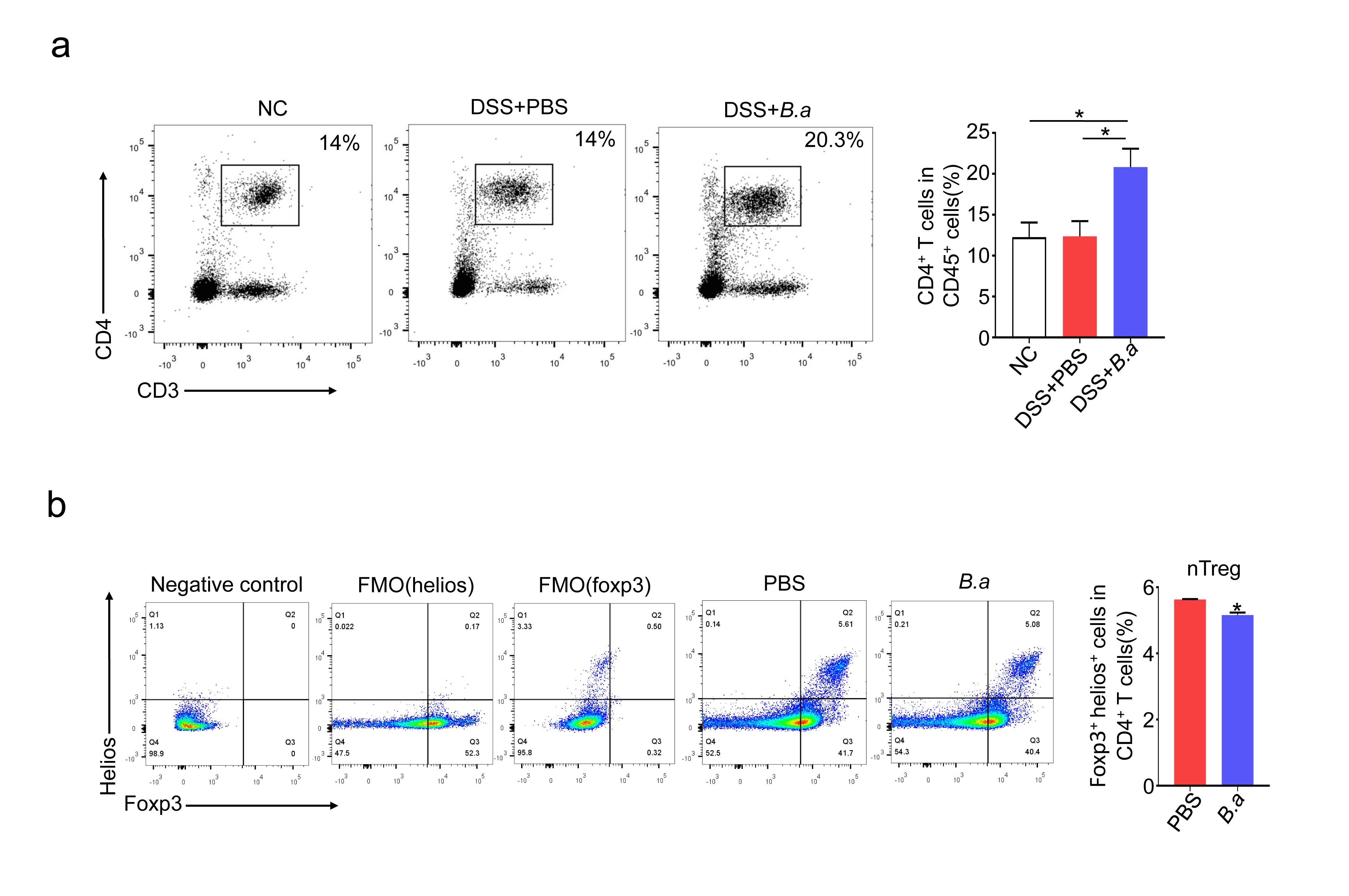

Supplement: Supplemental Material [file KGMI_A_1826746_SM2661.zip › Supplementary information/Supplementary Figure 2.tif]

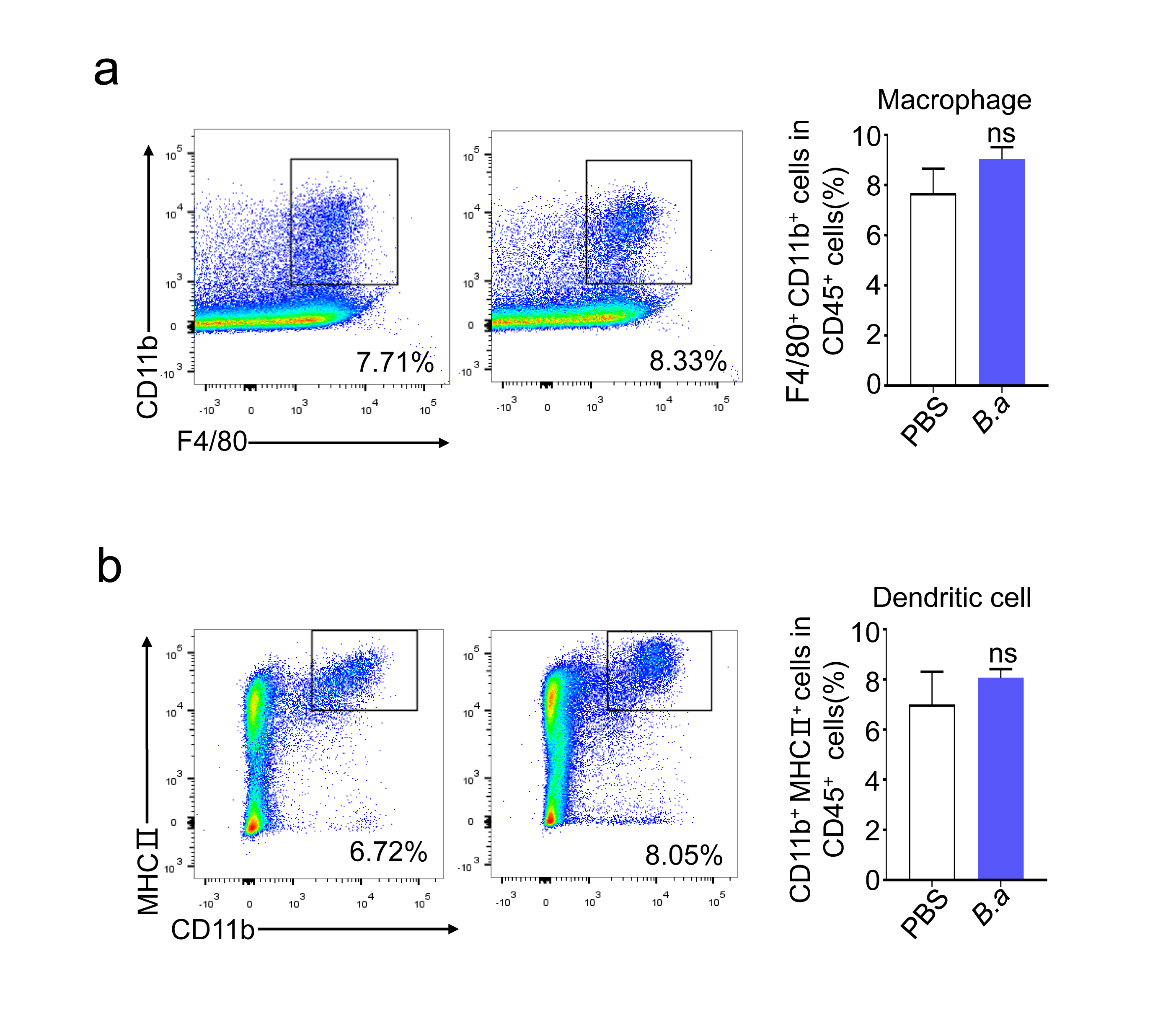

Supplement: Supplemental Material [file KGMI_A_1826746_SM2661.zip › Supplementary information/Supplementary Figure 3.tif]
